# Mother’s obesity and high child’s waist circumference are predictive factors of severe child’s obesity: an observational study in French Guiana

**DOI:** 10.1186/s12887-018-1158-z

**Published:** 2018-06-09

**Authors:** Falucar Njuieyon, Emma Cuadro-Alvarez, Elise Martin, Noémie Lachaume, Yajaira Mrsic, Fanny Henaff, Chimène Maniassom, Antoine Defo, Narcisse Elenga

**Affiliations:** 1 Department of Pediatric Medicine and Surgery, Cayenne Hospital, Rue des flamboyants, BP 6006, 97306 Cayenne Cedex, French Guiana; 2 Department of Pediatric Medicine and Surgery, Regional Hospital, Rue des Flamboyants BP 6006, 97306 Cayenne Cedex, French Guiana

**Keywords:** Mother’s obesity, Waist circumference, Predictive factors, Childhood obesity, Observational study, French Guiana

## Abstract

**Background:**

This study aims to describe the predictive factors of severe obesity in children followed in French Guiana.

**Methods:**

In this observational study, the patients from the French Guianese Childhood Obesity Group database were prospectively included**,** after giving a statement of patient’s non opposition.

**Results:**

Our group classifications revealed that 36 of 150 (24%) participants were classified as being metabolically abnormal obesity“ (MAO), while 114 of 150 (76%) were categorized as metabolically normal obesity” (MNO). MAO-patients were older. Their mothers had more severe obesity. We also observed that their systolic blood pressure was higher. The median Z-score BMI of children with MAO was 4, 9 [4, 05–5, 38], which shows a more obese condition than the MNO group. The median waist-to-height ratio (WTHR) of our study population was high, either 0.63 [0.54–0.59]. No significant differences in the term of pregnancy, father’s obesity, gender, birth weight, feeding, diastolic blood pressure and WTHR were found between the two groups. The predictors of MAO status, after adjusting for age and sex, were mother’s obesity and high child’s waist circumference.

Among the comorbidity, there were two Down syndrome, one Cornelia de Lange syndrome, one Nephrotic Syndrome and one Epilepsy. The leptin hormone and insulin levels were higher in MAO than in MNO, while 25-OH D-vitamin was higher in MNO.

**Conclusion:**

This study indicates the need to incorporate waist circumference into routine clinical practice, in addition to traditional measures of weight, height, body mass index and waist-to-height ratio.

## Background

Childhood obesity has continued to increase over the last 30 years in the world and in France. The field observations show that the prevalence of overweight and obesity among children in French Guiana is almost twice that in metropolitan France [1]. Cases of childhood type 2 diabetes associated with obesity are also observed [1].

“Overweight and obese children are likely to stay obese into adulthood and more likely to develop noncommunicable diseases like type2 diabetes or hypertension at a younger age” [2]. The fight against obesity needs multicomponent interventions including lifestyle changes reduced caloric intake, decreased sedentary behaviour and increased physical activity [3, 4]. These interventions have also been proved successful for the prevention and treatment of child and adolescent obesity [5, 6]. French public health ministry policy for fight against childhood obesity is focused on therapeutic education programs [7]. In French Guiana, all newly diagnosed childhood type2 diabetes are severely obese [1]. Prevention of childhood obesity therefore needs special attention and high priority.

French Guiana is an overseas department and region of France, located on the north Atlantic coast of South America in the Guianas. It borders Brazil in the east and south, and Suriname in the west. Its 83,534 km^2^ area has a very low population density. In January1^st^ 2017, French National Institute for Statistics and Economic Studies (INSEE) estimated the population of French Guiana to be 279,933 people. Among the characteristics of this population were its youthfulness (44% below the age of 20), its multigene rational crossbreeding and the fact that the population is also facing demographic transition [8].

Cayenne Hospital is the main referral hospital in French Guiana. The day hospital of the pediatric department leads many authorized therapeutic education programs among which that for the fight against childhood obesity. More than 350 children with chronic diseases are thus involved in different therapeutic education programs. This study aims to describe the predictive factors of severe obesity in children followed in French Guiana.

## Methods

### French Guianese childhood obesity group (GuiChOG)

Since 2010, the GuiChOG has been created and is being held in the pediatric department of Cayenne Hospital. This group is part of the therapeutic education programs against obesity in children conducted in the pediatric day hospital. The high prevalence of overweight and obesity in French Guianese children, its close association with type 2 diabetes [1], motivated the group to understand the determinants of obesity among children in this specific population. Tips for overweight or obese children are included consecutively in this group. A GuiChOG Excel database is prospectively extracted from a medical file of these obese children after the completion of the first diagnostic consultation performed according to the High Authority of Health (HAS) recommendations for the French National Nutrition and Health Program (PNNS) [9].

#### Ethical statements

All data were collected, after certification of a written patient’s non opposition. All underage participants had written informed consent provided on their behalf by their parent/legal guardian. According to the European regulation, French observational studies from data obtained routinely, from patient health-care records, do not need the approval of an ethics committee [10]. These anonymized data issued from medical records were analyzed, which was authorized according to the Regulatory authorities (Commission Nationale Informatique et Libertés (CNIL) number 2046957 v 0.

#### Study population

Most consultations for overweight or obesity originated from general practitioners, school nurses or parents on their own. Each consulting child has a physical exam done in the same box and additional analyzes done at the day hospital of the pediatric department. Anthropometric assessments of children with the parent present were obtained out of the physical exam. Blood pressure (BP) was obtained prior to hormonal and metabolic analyzes. The number of children progressively increased since the first inclusion in the GuiChOG. After six years, 150 children completed the anthropometric, hormonal and metabolic evaluation.

#### Measurements

The same pediatrician performed the clinical measurements in children involved in the study. The height was measured to the nearest 0.1 cm using a wall mount mechanical Seca 206 bodymeter. The Waist circumference (WC) was measured midway between the lowest border of rib cage and the upper border of iliac crest, at the end of normal expiration, using a Seca 201CM Ergonomic Circumference Measuring Tape. The supine length was measured to the nearest 0.5 cm with a standardized length board consisting of a fixed board for the infant’s head and a movable board allowing feet to be placed perpendicular to the longitudinal axis of the infant. The weight was measured to the nearest 0.1 kg using a medical digital balance (Seca Mechanical Floor Scales - Model 762). The body mass index (BMI = weight/length^2^) was calculated. The weight and height of parents present during the visit was measured and BMI calculated. BMI was converted into z-scores to adjust for age and sex using the French references curves (Rolland-Cachera, Sempé) [11, 12]. We also performed the waist-to-height ratio (WTHR) measurement as it is useful as a screening tool for metabolic problems related to obesity because of its convenience [13].

The blood pressure was measured on the right arm of seated subjects after 5 min of rest, using a GE Dinamap ProCare Auscultatory 400 Vital Signs Monitor and an appropriately sized arm cuff. Three measurements were undertaken per participant. For each patient, the retained blood pressure value for this study was the mean of three measurements [14]. Above 4 years of age, glucose tolerance was assessed using an oral glucose tolerance test (OGTT) performed with the administration of 1.75 g of glucose solution per kilogram of body weight (without exceeding 75 g per dose, whatever the weight), after an overnight fast [15]. The blood samples were drawn at 0, 30 and 120 min for measurements of glucose and insulin.

#### Assays

Blood analyses were performed on a venous cord blood sample obtained after an overnight fast. Glucose was measured immediately whereas samples for hormonal analysis were quickly centrifuged and serum was separated and stored at − 80 °C until analysis. Serum insulin was measured by an IRMA kit (BI-INS-IRMA) from Cis Bio international (Gifsur-Yvette, France). Cross-reactivity with pro insulin and derived metabolites was less than 1%. Assay sensitivity was 3.0 pmol/L. Serum leptin was measured using a specific radioimmunoassay (Linco research, St Charles, USA). Sensitivity of the assay is 0.4 ng/ml. Intra- and inter- assay coefficients of variation are 5.2 and 8.7% respectively at 2.3 ng/ml. Insulin sensitivity was assessed from fasting insulin and glucose levels using the index QUICKI (Quantitative insulin sensitivity check index) as 1/(log (fasting insulin) + log (fasting glucose) [16].

#### Definition of overweight and obesity

Body mass index (BMI) is a measure used to determine childhood overweight and obesity. Overweight is defined as a BMI at or above the 85th percentile and below the 95th percentile for children and teens of the same age and sex [17]. Obesity is defined as a BMI at or above the 95th percentile for children and teens of the same age and sex [17].

#### Definitions of metabolic risk

For children, metabolic syndrome can be diagnosed with abdominal obesity (using waist circumference percentiles) and the presence of two or more other clinical features (elevated triglycerides, low HDL-cholesterol, high blood pressure, increased plasma glucose) [18]. To examine the presence and predictors of Metabolically normal obesity (MNO), we applied a more clinically relevant classification, in which participants were dichotomized based on the presence/absence of the following five traditional criteria of metabolic syndrome: obesity, hyperglycemia, atherogenic dyslipidemia, low HDL-cholesterol and hypertension (MNO: 0; metabolically abnormal obesity (MAO): ≥2criteria) [19, 20].

#### Statistical analysis

Statistical analyses were performed using STATA software version 13 (*Stata Statistical Software: Release 13*. College Station, TX: StataCorp LP) with statistical significance set at *P* < 0.05. Means, SDs, and ranges were calculated for all continuous variables. Independent samples *t* tests were used to compare continuous variables between groups. Multivariable logistic regression was used to examine the association between each of the variables and metabolic unhealthy status with adjustment for age and sex. Because there were no sex interactions, all analyses were conducted collapsed across sex. Subsequently, each of the strongest independent predictors of MAO within three main categories of variables: *1*) adiposity (weight, BMI, BMI percentile, BMI z-score, and waist circumference); and *2*) PA-related (moderate PA, very hard PA,) were entered into a logistic regression model with adjustment for age and sex. To facilitate comparisons between variables, odds ratios (ORs) were expressed per SD units. All analyses were adjusted for sex and age, except when BMI z-score was included in the models since this variable already adjusts for interindividual differences in sex and age. The final model included variables that were significantly associated with the severe obesity in a single covariable analysis.

## Results

Our group classifications revealed that 36 of 150 (24%) participants were classified as being MAO, while 114 of 150 (76%) were categorized as MNO (Table 1). Patients with MAO were older. Their mothers had more severe obesity. We also observed that their systolic blood pressure was higher. The median Z-score BMI of children with MAO was 4, 9 [4, 05–5, 38], which shows a more obese condition than the MNO group. The median waist-to-height ratio (WTHR) of our study population was high, either 0.63 [0.54–0.59]. No significant differences in the term of pregnancy, father’s obesity, gender, birth weight, feeding, diastolic blood pressure and WTHR were found between the two groups (Table 1). The predictors of MAO status, after adjusting for age and sex, were mother’s obesity and high child’s waist circumference (Table 1). We described comorbidity in 12 patients (Table 2). Among them, there were two Down syndrome, one Cornelia de Lange syndrome, one Nephrotic Syndrome and one Epilepsy. Table 3 showed that leptin hormone and insulin levels were higher in MAO than in MNO, while 25-OH D-vitamin was higher in MNO. There was no statistically significant difference of urinary free cortisol between the two groups.

**Table 1:** Comparison of demography, anthropometry and clinical characteristics of obese children

Characteristics	Metabolically Normal Obesity *n* = 114 (%)	Metabolically Abnormal Obesity *n* = 36 (%)	*p*-value	p*
Age (years, median, range)	8,85 (5,92–11,10)	11,12 (9,93–13,36)	< 0,001	
Sex			0.09	
Boys	63 (55)	14 (39)		
Girls	51(45)	22 (61)		
Birth weight (Kg, median, range)	3.310 (3.020–3.560)	3.175 (2.940–3.580)	0.5	
Comorbidity			0.9	
No	104 (91)	33 (92)		
Yes	10 (9)	3 (8)		
Mother’s obesity (*n* = 123)			0.003	0,02
Nonobese (BMI < 25 Kg/m2)	19 (20)	1 (4)		
Overweight (25 < BMI < 30 Kg/m2)	32 (34)	4 (14)		
Obese (BMI?30/Kg/m2)	44 (46)	23 (82)		
Father’s obesity (*n* = 68)			0.6	
Nonobese (BMI < 25 Kg/m2)	10 (18)	3 (25)		
Overweight (25 < BMI < 30 Kg/m2)	21 (38)	4 (33)		
Obese (BMI?30 /Kg/m2)	25 (45)	5 (42)		
Term pregnancy (n = 123)				
Full-term birth	91 (97)	28 (97	0.9	
Premature birth	3 (3)	1 (3)		
Feeding (*n* = 129)			0.1	
Breastfeeding	25 (26)	4 (13)		
Formula or mixed	73 (74)	27 (87)		
BMI (Z-score, median, range)	4,42[3,94–5,28]	4,9[4,05–5,38]	< 0,001	
Overweight or obesity				
Overweight	6 (5)	0 (0)	0.6	
Obesity	108 (95)	36 (100)		
Systolic blood pressure (mmHg, median, range)	111 (106–121)	133.5 (123.5–138.5)	0.001	
Diastolic blood pressure (mmHg, median, range)	70 (66–77)	79.5 (72.5–84.5)	0.06	
Waist circumference (cm, median, range)	85 (78–96)	103 (94–109)	< 0.001	< 0.001
Waist-to-height ratio	0.62 [0.58–0.67]	0.66 [0.61–0.69]	0.3	

p*obtained after a multivariate analysis

**Table 2:** Comorbidities in obese children

Comorbidities	Number
Asthma and allergy	2
Tyrisomy 21	2
Type 2 diabetes	1
Hemoglobin Korle Bu	1
Sickle cell HbSC disease	1
Cornelia de Lange syndrome	1
Nephrotic Syndrome	1
Epilepsy	1
Psychomotor retardation	1
Dysmorphic syndrome	1

**Table 3:** Biological characteristics of obese children

Characteristics	Metabolically Normal Obesity *n* = 114 (%)	Metabolically Abnormal Obesity *n* = 36 (%)	*p*-value
Triglycerids (mmol/L) median, range	0.76 (0.62–1,27)	0.99 (0,70–1,58)	0.2
HDL cholesterol (mmol/l) median, range	1.19 (0.99–1.36)	1.22 (0.96–1.39)	0.5
Total cholesterol (mmol/l) median, range	4.1 (3.64–4.70)	4.07 (3.50–4.62)	0.7
HbA1C (%)	5.2 (4.9–5.35)	5.5 (5.1–5.8)	0.1
Leptin hormone (ng/ml) median, range	26.95 (17.82–40.96)	35.6 (28.85–48.75)	0.02
IGF1 (ng/ml) median, range	221 (185–267.7)	276.2 (237.9–325.7)	0.3
IGFBP3 (mg/l) median, range	4.6 (3.7–25.71)	4.8 (4.7–5.4)	0.4
Insulin level (μU/ml) median, ragne	12.8 (6.9–20.7)	24.4 (15.65–38.05)	0.01
Glycemia (mmol/l) median, range	4.6 (4.3–5.1)	4.8 (4.5–5.05)	0.3
25 OHD Vitamin (μg/l) median, range	28.2 (24–33)	25 (22–31)	0.03
Urinary free cortisol l(nmol/24 h) median, range	43 (21–60)	42 (24.5–67)	0.7

*HDL* high density lopoprotein

## Discussion

The high prevalence of pediatric obesity highlights the importance to understand its associated factors in order to offer a multidisciplinary weight management care for children with obesity.

It has been described a correlation between sedentary behaviour (SED) in children and elevated risk of obesity because of parental obesity [21–23]. It is also known that childhood obesity is connected with familial and environmental factors, including incorrect eating habits [24–26].

Our study confirms that the child’s obesity is often related to that of the parents, especially that of the mother [27–33]. Indeed, maternal obesity just before pregnancy was associated with more than triple the likelihood of severe childhood obesity [34]. Mothers play a crucial role in the family fabric as they are a model for their children. Thus, the prevention of obesity must be done by supporting mothers to build a healthy home environment. [35]. There are certainly genetic factors, but dietary habits also play a major role. For example, one study found that consuming fruits, even from children whose mothers were very obese during pregnancy, reduced by three, the risk of obesity [36]. We also highlight the need of monitoring the waist circumference, in order to prevent the worsening of obesity. Waist circumference for age and gender is used to define abdominal obesity [37]. WC is the simplest and most widely accepted clinical measure for measuring central pubertal obesity. It is a non-invasive and easy to perform method. In young children, WC is a better estimate of body fat percentage, after sex and age adjustment. According to the literature data [38], among adolescents, the waist circumference tends to increase with age in both girls and boys. This is a phenomenon expected during puberty, which represents a critical period for the development and distribution of body fat. At equal ages, boys often have higher waist circumference values than girls. This is probably explained by the distribution of adipose tissue that is different in boys and girls. Boys are mostly faced with an overload android, with accumulate fat on the upper body, while in girls, fat accumulates mostly on the lower body. Thus, waist circumference measurement can be used to determine the risk profile of metabolic syndrome and cardiovascular disease.

The same is true for risk factors for cardiovascular disease in children, where WC is a better predictor than BMI [39, 40]. In our study, breastfeeding was not associated with child’s BMI at this age-group. Even though it was high in our study population, the WHTR showed no significant relationship with the MAO. In agreement with other studies, WHTR is less useful in classifying children’s obesity status than BMI or WC [41, 42].

Children with MAO had significantly lower mean 25(OH)D levels than those with MNO. Several mechanisms could explain the relationship between Vitamine D deficiency and obesity. These mechanisms include the dilution or deposition of ingested or dermally synthesized Vitamin D in high-volume fat compartments, reducing its bioavailability [43, 44], a decrease in the exposure to solar UV radiation and a decrease of the external activity of the cutaneous vitamin D synthesis [45]. Leptin hormone and insulin levels were higher in MAO than in MNO. Authors have reported a relationship between Vitamin D, insulin resistance and leptin level. High leptin levels increase the expression of pro-inflammatory and pro-angiogenic cytokines [46]. However, vitamin D deficiency is associated with chronic inflammation and may predispose to insulin resistance [47–49].

Our study had several strengths, such as the fact that similar studies have not been carried out previously in Guianese children. Trained health professionals who used the same anatomical sites and measurement tools collected anthropometric data. In addition, the results are likely to be representative of severe obese children in Cayenne because the BMI data were collected over a specific recent period within the local pediatric unit only.

Our study presents also some limitations, among which the lack of information on the effect of the pubertal state on the anthropometric indices. Children with MAO were significantly older and probably more sexually mature than those with MNO, which might have affected the fat distribution and biased the anthropometric results. The monocentric character of the study and the low power do not allow the generalization of these data throughout French Guiana.

These findings suggest the value of early and careful monitoring of BMI and WC in order to identify in time the children most at risk of severe obesity and metabolic syndrome in adolescence. Although further studies on the risk factors for severe obesity are needed, the factors described in our study could be considered in screening, monitoring, and interventions to reduce severe childhood obesity.

## Conclusion

Our data can be used to inform clinicians about the heterogeneity of pediatric obesity. They indicate the need to incorporate waist circumference into routine clinical practice, in addition to traditional measures of weight, height, BMI and WHTR.

## Abbreviations

BMI: Body mass index
BP: Blood pressure
CNIL: Commission Nationale Informatique et Libertés
GuiChOG: French Guianese Childhood Obesity Group
HAS: High Authority of Health
HDL: High density lipoprotein
INSEE: French National Institute for Statistics and Economic Studies
MAO: Metabolically abnormal obesity
MNO: Metabolically normal obesity
OGTT: Oral glucose tolerance test
OR: Odds ratios
PNNS: French National Nutrition and Health Program
QUICKI: Quantitative insulin sensitivity check index
SD: Standard deviation
SED: Sedentary behaviour
WC: Waist circumference
WTHR: Waist-to-height ratio
